# Impact of lowering the risk threshold for statin treatment on statin prescribing: a descriptive study in English primary care

**DOI:** 10.3399/bjgp20X713057

**Published:** 2020-10-06

**Authors:** Alexander Pate, Richard Emsley, Tjeerd van Staa

**Affiliations:** Centre for Health Informatics, University of Manchester, Manchester.; Department of Biostatistics and Health Informatics, King’s College London, London.; Centre for Health Informatics, University of Manchester, Manchester.

**Keywords:** cardiovascular diseases, health services research, hydroxymethylglutaryl-CoA reductase inhibitors, primary health care

## Abstract

**Background:**

In 2014, the National Institute for Health and Care Excellence (NICE) changed the recommended threshold for initiating statins from a 10-year risk of cardiovascular disease (CVD) of 20% to 10% (Clinical Guideline 181), making 4.5 million extra people eligible for treatment.

**Aim:**

To evaluate the impact of this guideline change on statin prescribing behaviour.

**Design and setting:**

A descriptive study using data from Clinical Practice Research Datalink (CPRD), a primary care database in England.

**Method:**

People aged 25–84 years being initiated on statins for the primary prevention of CVD were identified. CVD risk predictions were calculated for every person using data in their medical record (calculated risks), and were extracted directly from their medical record if a QRISK score was recorded (coded risks). The 10-year CVD risks of people initiated on statins in each calendar year were compared.

**Results:**

The average ‘calculated risk’ of all people being initiated on statins was 20.65% in the year before the guideline change, and 20.27% after. When considering only the ‘coded risks’, the average risk was 21.85% before the guideline change, and 18.65% after. The proportion of people initiating statins that had a coded risk score in their medical record increased significantly from 2010–2017.

**Conclusion:**

Currently available evidence, which only considers people with coded risk scores in their medical record, indicates the guideline change had a large impact on statin prescribing. However, that analysis likely suffers from selection bias. This new evidence indicates only a modest impact of the guideline change. Further qualitative research about the lack of response to the guideline change is needed.

## INTRODUCTION

In July 2014, the National Institute for Health and Care Excellence (NICE) changed the recommended threshold for initiating statin treatment for primary prevention of cardiovascular disease (CVD) from a 10-year CVD risk of 20% to 10% (Clinical Guideline [CG] 181).^1^ This decision came alongside huge debate in academic and clinical literature, as lowering thresholds could have a huge impact on clinical practice.^2^^–^^4^ It was estimated that the guideline change would make a total of 11.8 million people in England (37% of adults aged 30–84 years) eligible for statins,^5^ and was met with opposition by a group of leading doctors.^6^ NICE estimated that an additional 4.5 million people would be eligible for statins, preventing up to 28 000 heart attacks and 16 000 strokes each year.^7^ Without an increase in statin prescribing in people with 10-year CVD risks between 10–20%, this number of extra heart attacks and strokes would not be prevented.

To the authors’ knowledge, only one study has assessed the impact of this major guideline change in practice (see section called ‘Impact of NICE guidance’ in Finnikin *et al*).^8^ In England, the QRISK3^9^ (previously QRISK2)^10^ risk prediction model is recommended by NICE for calculating the 10-year risk of a CVD event to guide treatment decisions for the primary prevention of CVD. The study analysed people who were initiated on statin treatment and had a QRISK2 score recorded in their electronic health record (EHR). It found that the average risk score of people receiving statins dropped from 23.06% before the guideline change to 19.28% after.^8^ This provides evidence that the guideline change was impactful, and the results are quoted in the NICE impact report for CVD prevention.^11^ The same study also reports, however, that, since 2012, 72.9% of people initiated on statins did not have a QRISK2 score recorded.

The aim of the present study was to evaluate the impact of reducing the risk threshold from 20% to 10% by analysing the risks of all people being initiated on statins for primary prevention of CVD. The study also replicates the analysis carried out by Finnikin *et al*,^8^ considering only people with a QRISK score in their medical record.

## METHOD

### Cohort definition

This project used data from the Clinical Practice Research Datalink (CPRD).^12^ These data were linked with data from Hospital Episodes Statistics (HES)^13^ and the Office for National Statistics (ONS)^14^ for identifying CVD events. Linkage to HES restricts this dataset to England only. Two cohorts were defined: a primary prevention cohort and a statin initiation cohort. The primary prevention cohort consisted of people aged 25–84 years with no history of CVD (composite outcome of coronary heart disease, ischaemic stroke, or transient ischaemic attack) or statin use. The cohort entry date was defined as the latest of 25th birthday, 1 year permanently registered in CPRD, or 1 January 1998. People were excluded if they had a CVD event or statin prescription before their cohort entry date (for code lists see Supplementary Box S1). People were censored at the earliest date of transferred out of practice, last data collection for practice, CVD event, death, or 31 December 2017.

**Table:** How this fits in

In 2014, the National Institute for Health and Care Excellence (NICE) introduced new guidelines reducing the risk level at which people become eligible for statin treatment to prevent cardiovascular events, from a 10-year risk of 20% to 10%. In the current literature, only one study has evaluated this guideline change in practice, and the results indicated that the guideline change has led to a large change in prescribing behaviour. However, this study indicates that the change in prescribing behaviour may be much smaller than is currently thought. The findings are important for NICE to understand that this guideline may not be getting widely applied in practice. It is also important for GPs to provide feedback on why this may be the case, and whether the guideline is clinically acceptable or not. Such discussions could lead to the development of guidelines that will be more widely adopted in practice.

An individual from the primary prevention cohort was included in the statin initiation cohort on the date of their first statin prescription if this first statin prescription was issued ≥1 year after the start of follow-up.

### Statin initiation rate

The primary prevention cohort was used to calculate the statin initiation rate each year. For each calendar year the total number of statin initiations was calculated, as was the total number of days of follow-up. Follow-up for each person stopped either when they were censored or initiated on statin treatment. Calendar years ran from 1 July each year, to match the date at which the guidelines were changed (July 2014). The final period (2017–2018) finished on 31 December 2017, and as such was only 6 months long.

### Comparisons of risks of people initiated on statins each year

For each individual in the statin initiation cohort, all the predictors required to generate a QRISK3 score were extracted (for a full list of variables, code lists, information on variable derivation, the amount of missing data, and details of the imputation process see Supplementary Boxes S1 and S2). The 10-year CVD risk of each person at statin initiation was then calculated using QRISK3; an R package was used for this.^15^ These are referred to as the ‘EHR-derived risks’.

Where recorded, coded QRISK scores were extracted directly from the EHR if they were within 180 days before or 30 days after the first statin prescription (for code list see Supplementary Box S1). These risk scores are referred to as ‘coded risks’, and are used to replicate the analysis by Finnikin *et al*.^8^ The coded risks will have been calculated using a mix of iterations of the original QRISK^16^ algorithm and multiple versions of the QRISK2 algorithm.

The following analyses were carried out using both the EHR-derived and coded risks. The average risk of people initiated on statins in each calendar year was calculated. Intervals ran from 1 July, to match the date of the threshold change, which was July 2014. The proportion of people initiated on statins each year that were classified as low risk (<10%), intermediate risk (10–20%), or high risk (>20%) were calculated.

The agreement between the EHR-derived risks and coded risks was evaluated using scatter plots. This was done to check agreement between the EHR-derived and coded risk scores. A higher level of agreement would provide support that the analysis based on the EHR-derived risks is valid, given that the coded risks can be viewed as the gold standard. By using scatter plots, agreement was compared on the most granular level possible (that is, does the EHR-derived risk match the coded risk in the database for each individual person?).

## RESULTS

The primary prevention cohort included 3 892 603 individuals (51% female). The statin cohort consisted of 351 553 individuals (47% female). For the demographics of the statin cohort, see Supplementary Table S1.

**Figure 1.**
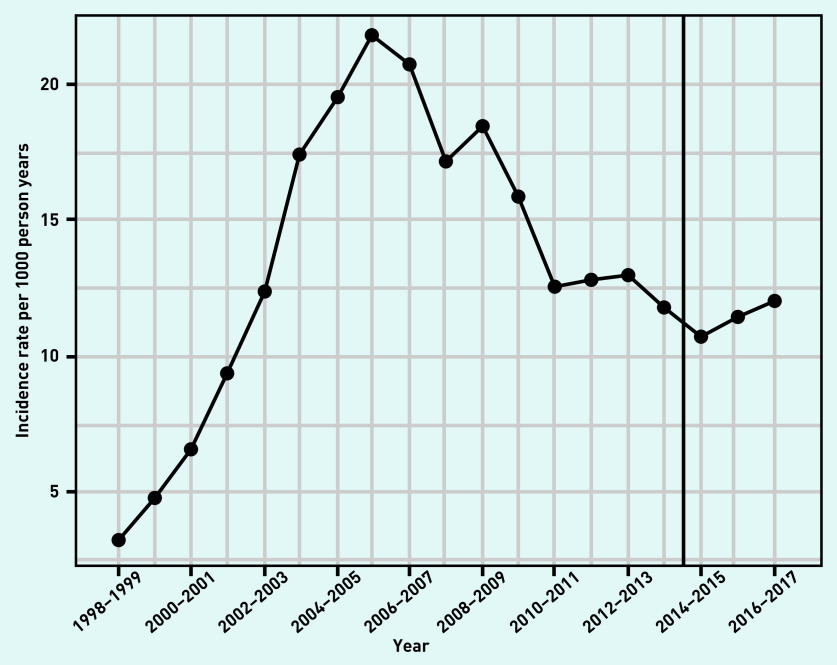
***Statin initiation rate in each year***

The statin initiation rate per 1000 person years by calendar time is presented in Figure 1. Visible in the graph is a peak of 21.79 in 2005, and a drop until 2010–2011, when the incidence rate flattens out at around 12.53 in 2010–2011.

The number of people initiated on statins each year is provided in Table 1, as well as the number of those statin initiations that have an associated coded score. Before 2010–2011 <5% of the statin initiations had an associated coded risk score. After this the proportion increases to 66.29% by 2017–2018.

**Table 1.** Number of people initiated on statins each year, and number of those who had an associated coded QRISK score

**Date**	**Follow-up, person years**	**Initiated, *N***	**With coded score, *N***	**Proportion coded, %**
**1998–1999**	1 090 072.9	3510	26	0.74
**1999–2000**	887 549.2	4240	66	1.56
**2000–2001**	1 141 713.0	7498	232	3.09
**2001–2002**	1 318 576.6	12 335	450	3.65
**2002–2003**	1 449 309.0	17 908	457	2.55
**2003–2004**	1 547 360.7	26 959	322	1.19
**2004–2005**	1 563 126.1	30 529	272	0.89
**2005–2006**	1 588 051.6	34 604	390	1.13
**2006–2007**	1 591 314.4	32 967	316	0.96
**2007–2008**	1 598 293.4	27 432	211	0.77
**2008–2009**	1 601 472.2	29 554	501	1.70
**2009–2010**	1 569 415.3	24 883	1053	4.23
**2010–2011**	1 513 887.1	18 972	1156	6.09
**2011–2012**	1 453 955.3	18 622	2314	12.43
**2012–2013**	1 402 210.8	18 181	3219	17.71
**2013–2014**	1 245 691.7	14 689	3831	26.08
**2014–2015**	1 021 942.4	10 938	4677	42.76
**2015–2016**	749 647.7	8572	5012	58.47
**2016–2017**	540 323.3	6511	4188	64.32
**2017–2018**	233 582.6	2649	1756	66.29

**Figure 2.**
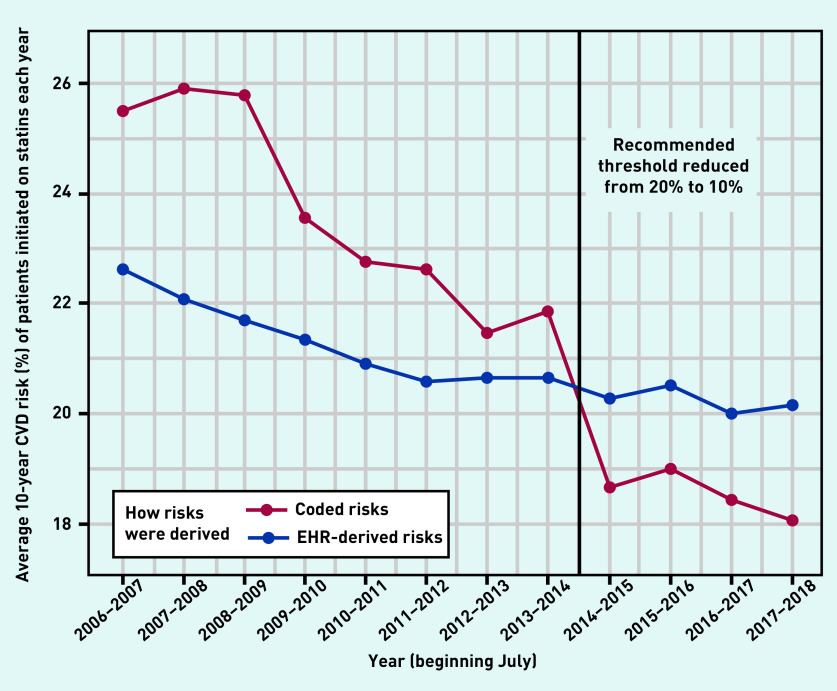
***Average 10-year CVD risk of people initiated on statins in each year. CVD = cardiovascular disease. EHR = electronic health record.***

Figure 2 plots the average EHR-derived risk and average coded risk of people being initiated on statins each year. The latter is restricted to those who had a coded risk score available. There is no clear change to the average EHR-derived risk of people being initiated on statins from 2013–2014 (20.65%) to 2014–2015 (20.27%), which are the years before and after the guideline change. There is, however, a drop in the average coded risk from 21.85% to 18.65%.

**Figure 3.**
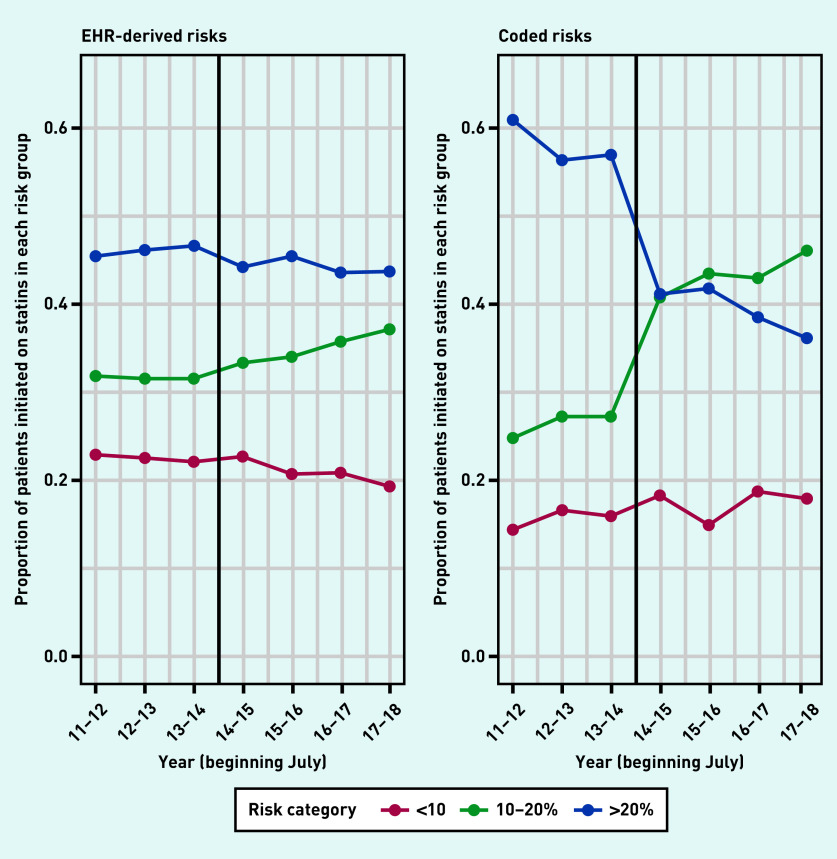
***Proportion of people initiated on statins each year that belong to each risk category (<10%, 10–20%, >20%). EHR = electronic health record.***

Figure 3 shows the proportion of people initiated on statins each year that belong to each risk category. For the EHR-derived risk scores there is a steady increase in the proportion of people in the 10–20% risk group from 2013–2014 onwards. However, this happens mostly at the expense of people from the <10% group, as well as some from the >20% group. For the average coded risk score, there is a sharp increase in the proportion of people in the 10–20% risk group, which comes at the expense of people in the >20% group.

**Figure 4.**
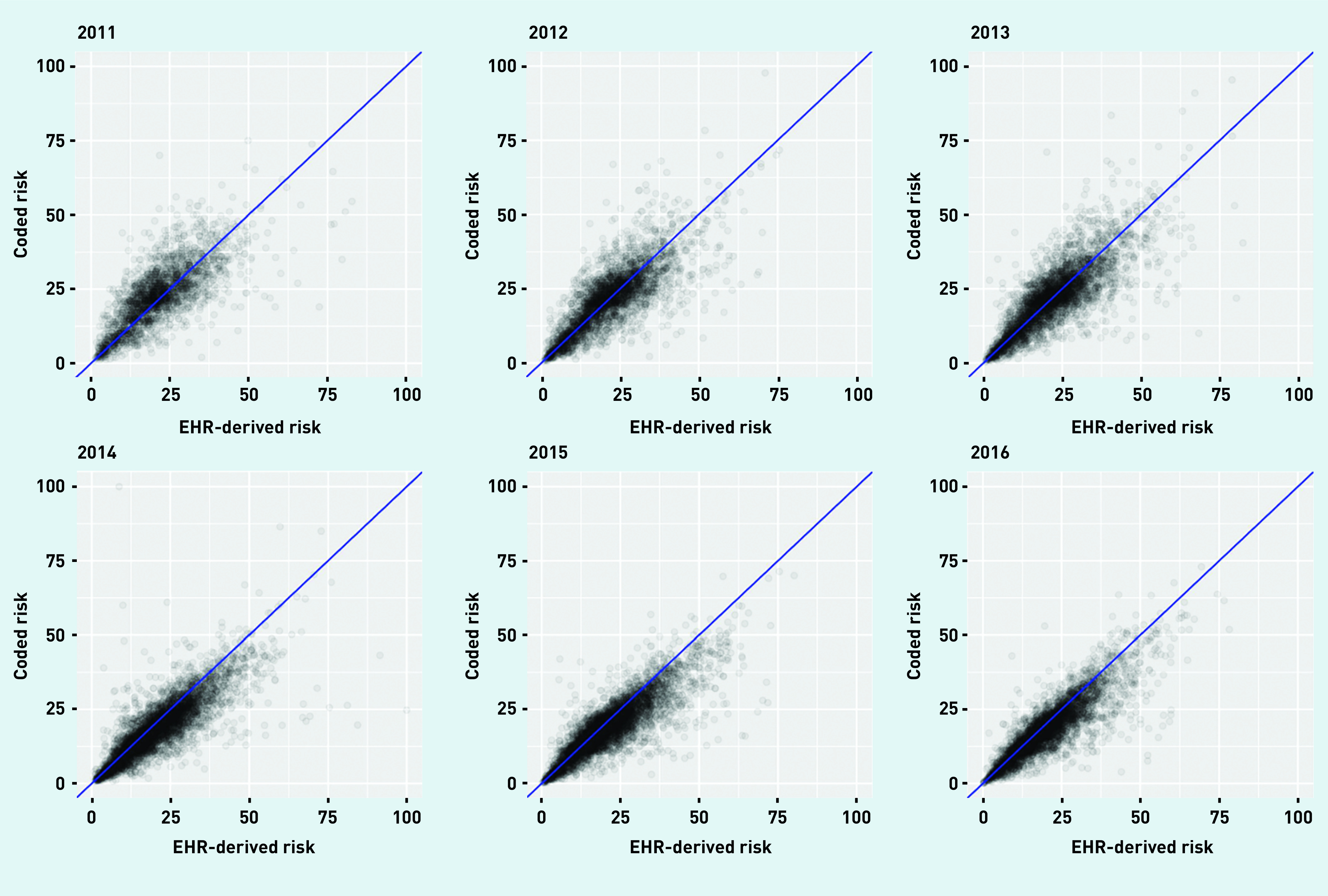
***EHR-derived risks plotted against coded risks, stratified by year of statin initiation. Blue line = 1:1 correlation. EHR = electronic health record.***

Figure 4 plots the EHR-derived risks against the coded risk scores for each individual stratified by year, with a blue line added to illustrate perfect correlation. Overall, there is a strong positive relationship between the two, although there are quite large levels of variation either side of perfect agreement. Also, from 2014 onwards there is more consistent overprediction of the EHR-derived algorithm compared with the coded risk scores.

## DISCUSSION

### Summary

There was a large reduction in the average coded risk of people initiated on statins, which closely matches the currently available evidence.^8^ When viewed in isolation, the reduction in the average coded risk score (Figure 2) and the change of proportion in each risk category (Figure 3) indicate a significant change in clinical practice. NICE has quoted this evidence in their impact report.^11^ However, because the coded risk analyses only consider the subgroup of people with a coded risk score, this analysis is at risk of cohort selection bias, as the subgroup may not be representative of all people initiated on statins. This risk is exacerbated by the increasing proportion of people initiated on statins that have a coded risk score (Table 1). As this subgroup increases in size, unless risk scores are recorded at random this will have a significant impact on the average risk of this subgroup. Importantly, the changes in risk are driven by changes in for whom GPs are recording risk scores, rather than a change in who is receiving statins.

No change was found in the average EHR-derived risk of people being initiated on statins after the guideline change, and a small increase in the proportion of people that belonged to the 10–20% risk group was found. This analysis is not affected by the same selection bias, as it considers all people initiated on statins each year. Therefore with the extra data presented in this paper the authors believe the response to the guideline change is not as impactful as first thought.

### Comparison with existing literature

To the authors’ knowledge, only one other study has measured the impact of CG181 on clinical practice.^8^ This research is enhanced with an expanded analysis considering all people initiated on statins, their findings validated by replicating their analysis in CPRD. This study’s data indicate recording practices of GPs had a significant impact on the average coded risk. The proportion of people with a coded risk is small and increasing rapidly at this time (26.08% in 2013–2014 and 42.76% in 2014–2015). It is highly likely that the subgroup of people receiving a coded risk score was changing (it is unreasonable to assume that this increase in recording was happening at random), but the typical patient being initiated on statins was not. One hypothesis is that GPs became far more likely to calculate the risk of someone in the 10–20% range using a QRISK tool after the guideline change, but their prescribing behaviour remained the same.

No reduction was found in the average EHR-derived risk after the guideline change (Figure 2). Although this indicates the guideline change had no impact, considering all the results leads to a slightly different conclusion. This constant average risk appears to be caused by a combination of a small increase in 10–20% risk people initiated on statins, and a drop in low-risk (<10%) people. In Figure 3, a steady increase in the proportion of people in the 10–20% risk group can be seen, and a decrease in the other two groups (a larger decrease in the <10% risk group). It is possible that the guideline change has had an equal effect on preventing statin initiation in low-risk (<10%) people as it has on increasing statin initiation in the target 10–20% risk group, resulting in no change to the average EHR-derived risk.

The data agree with a modest impact of the guideline change, but the changes are far more subtle than would be concluded if looking at the currently available evidence. These findings are important, as the numbers in the widely quoted statistics — 4.5 million patients becoming eligible for statin treatment due to the guideline change^17^^–^^20^ and prevention of up to 28 000 heart attacks and 16 000 strokes each year^7^ — are likely far from being achievable.

### Strengths and limitations

This is the first study to evaluate the impact of the NICE guidance CG181 on the risks of all people receiving statins in England. The study cohort is large and results are likely generalisable to the English population as CPRD is representative of the UK in terms of age, sex, and ethnicity.^12^

There are two key limitations in this work. The first is the imperfect agreement between the EHR-derived risks and the coded risks, because the EHR-derived risks should represent the risks of individuals as closely as possible. Potential reasons for the disagreement between these and the coded risks are:

- the EHR-derived risks use the QRISK3 algorithm, whereas QRISK2 will have been used in practice over those years;
- this study used multiple imputation to impute missing data, whereas missing data is imputed using mean imputation when coded QRISK scores are generated by GPs;
- variables have been identified using code lists that may not perfectly match those used by the algorithm in practice; and
- this study considers coded risks within a window of 6 months before statin initiation, while patient data could have changed in that time.

Despite some disagreement, the relationship was strong enough that, if the people with a coded risk score were a random subset of all people initiated on statins, there would have been a large drop in the average EHR-derived risk after the guideline change (as there was in the average coded risk). This was not the case, indicating the likelihood of selection bias in the coded risk analyses. This necessitates the analysis using the EHR-derived risks, even if the estimated risks are not perfect.

The second limitation is that many practices left CPRD towards the end of this study, resulting in a risk of selection bias in the cohort if the drop-out was not at random. There is no reason to believe, however, that people from practices that dropped out were more or less likely to be initiated on statins. Furthermore, the results considering the coded risk scores were comparable with those of Finnikin *et al*,^8^ a study carried out in The Health Improvement Network database (https://www.the-health-improvement-network.com/en/),^21^ which has not suffered from this limitation.

### Implications for research

The change in NICE guidance appears to have had a small effect on statin prescribing by GPs. Given that NICE invests time and resources into developing these guidelines, it would be worthwhile for them to understand why there has been such little response. The authors propose a qualitative study with GPs and patients to assess the barriers to statin initiation for the primary prevention of CVD in people with 10–20% risk. A recent scoping review^21^ of the current literature regarding the use of statins to prevent CVD found only three studies specifically considering primary prevention of CVD, and that *‘it was difficult to interpret how doctors’ or patients’ attitudes would vary according to the risk profile of the individual patients’*. A systematic review provided a comprehensive review on patient attitudes towards taking statins;^22^ however, the majority of studies were looking at long-term adherence, as opposed to statin initiation. No studies have investigated specifically the willingness to initiate at a 10% or 20% threshold for primary prevention of CVD. A debate article published in 2016^23^ discusses patient attitudes to taking statins in light of the NICE guidance change, attributing the lack of uptake in lower-risk patients to transferability of evidence from research to practice and the potential for side effects. However, the evidence base^24^^–^^27^ for their findings pre-dates the large amount of pro-statin research that came about in 2013 that has fuelled the statin debate. The authors of that debate article also noted that *‘there is sparse literature regarding the views of GPs’*. Some qualitative research does exist in this area,^25^^,^^26^^,^^28^^–^^30^ but, again, no studies have been carried out in the wake of the NICE guidance, or on prescribing specifically at 10% compared with 20% risks.

## References

[1] National Institute for Health and Care Excellence (2014). Cardiovascular disease: risk assessment and reduction, including lipid modification CG181.

[2] Taylor F, Huffman M, Macedo A (2013). Statins for the primary prevention of cardiovascular disease. Cochrane Database Syst Rev.

[3] Abramson JD, Rosenberg HG, Jewell N (2013). Should people at low risk of cardiovascular disease take a statin?. BMJ.

[4] Collins R, Reith C, Emberson J (2016). Interpretation of the evidence for the efficacy and safety of statin therapy. Lancet.

[5] Hawkes N (2017). NICE guidelines could put 12 million UK adults on statins. BMJ.

[6] Wise J (2014). Open letter raises concerns about NICE guidance on statins. BMJ.

[7] National Institute for Health and Care Excellence (2014). Wider use of statins could cut deaths from heart disease. https://www.nice.org.uk/news/article/wider-use-of-statins-could-cut-deaths-from-heart-disease.

[8] Finnikin S, Ryan R, Marshall T (2017). Statin initiations and QRISK2 scoring in UK general practice: a THIN database study. Br J Gen Pract.

[9] Hippisley-Cox J, Coupland C, Brindle P (2017). Development and validation of QRISK3 risk prediction algorithms to estimate future risk of cardiovascular disease: prospective cohort study. BMJ.

[10] Hippisley-Cox J, Coupland C, Vinogradova Y (2008). Predicting cardiovascular risk in England and Wales: prospective derivation and validation of QRISK2. BMJ.

[11] National Institute for Health and Care Excellence (2018). NICEimpact cardiovascular disease prevention.

[12] Herrett E, Gallagher AM, Bhaskaran K (2015). Data Resource Profile: Clinical Practice Research Datalink (CPRD). Int J Epidemiol.

[13] Digital NHS (2019). Hospital Episode Statistics (HES). https://digital.nhs.uk/data-and-information/data-tools-and-services/data-services/hospital-episode-statistics.

[14] Office for National Statistics https://www.ons.gov.uk/.

[15] Li Y, Sperrin M, van Staa T (2019). R package ‘QRISK3’: an unofficial research purposed implementation of ClinRisk’s QRISK3 algorithm into R. F1000Res.

[16] Hippisley-Cox J, Coupland C, Vinogradova Y (2007). Derivation and validation of QRISK, a new cardiovascular disease risk score for the United Kingdom: prospective open cohort study. BMJ.

[17] Lambert C (2015). Everyday drugs: the great statins debate. New Scientist.

[18] Boseley S (2016). Statins prevent 80,000 heart attacks and strokes a year in UK, study finds. Guardian.

[19] BJCardio Staff (2014). New NICE guidance published. Br J Cardiol.

[20] Duerden M (2014). Low-cost statins offer clear benefits and low levels of risk. https://www.guidelinesinpractice.co.uk/cardiovascular/low-cost-statins-offer-clear-benefits-and-low-levels-of-risk/352523.article.

[21] Byrne P, O’Donovan Ó, Smith SM, Cullinan J (2019). Medicalisation, risk and the use of statins for primary prevention of cardiovascular disease: a scoping review of the literature. Health Risk Soc.

[22] Ju A, Hanson CS, Banks E (2018). Patient beliefs and attitudes to taking statins: systematic review of qualitative studies. Br J Gen Pract.

[23] Hassan Y, Ford J, Steel N (2016). Why are statin prescribing guidelines for primary prevention not always followed in primary care?. Br J Gen Pract.

[24] Wu J, Zhu S, Yao GL (2013). Patient factors influencing the prescribing of lipid lowering drugs for primary prevention of cardiovascular disease in UK general practice: a national retrospective cohort study. PLoS One.

[25] Kedward J, Dakin L (2003). A qualitative study of barriers to the use of statins and the implementation of coronary heart disease prevention in primary care. Br J Gen Pract.

[26] Fairhurst K, Huby G (1998). From trial data to practical knowledge: qualitative study of how general practitioners have accessed and used evidence about statin drugs in their management of hypercholesterolaemia. BMJ.

[27] Gale NK, Greenfield S, Gill P (2011). Patient and general practitioner attitudes to taking medication to prevent cardiovascular disease after receiving detailed information on risks and benefits of treatment: a qualitative study.. BMC Fam Pract.

[28] Krüger K, Leppkes N, Gehrke-Beck S (2018). Improving long-term adherence to statin therapy: a qualitative study of GPs’ experiences in primary care. Br J Gen Pract.

[29] Tanner RM, Safford MM, Monda KL (2017). Primary care physician perspectives on barriers to statin treatment. Cardiovasc Drugs Ther.

[30] Elisabeth A, Denig P, van Vliet T, Dekker JH (2009). Reasons of general practitioners for not prescribing lipid-lowering medication to patients with diabetes: a qualitative study. BMC Fam Pract.

